# Intravenous Injection of Sodium Hyaluronate Diminishes Basal Inflammatory Gene Expression in Equine Skeletal Muscle

**DOI:** 10.3390/ani13193030

**Published:** 2023-09-27

**Authors:** Savannah R. Gregg, Madison R. Barshick, Sally E. Johnson

**Affiliations:** School of Animal Sciences, Virginia Polytechnic Institute and State University, Blacksburg, VA 24060, USA; greggsr3@vt.edu (S.R.G.); mrbarshick@vt.edu (M.R.B.)

**Keywords:** equine, skeletal muscle, exercise, hyaluronic acid, inflammation

## Abstract

**Simple Summary:**

Horses subjected to an unaccustomed increase in exercise intensity can experience damage and subsequent acute inflammation within the skeletal muscle tissue that may hinder the performance of the horse by causing muscle swelling and soreness. Hyaluronic acid injection may suppress this exercise-induced inflammatory response by acting as an anti-inflammatory in the muscle. Adult Thoroughbred horses were injected intravenously with a commercial sodium hyaluronate product for 3 weeks prior to performing an exercise stress test. Muscle biopsy samples were obtained before and after the exercise stress test was performed. The results indicate that horses receiving the hyaluronic acid supplement had decreased expression of inflammatory genes within skeletal muscle, but no genes remained suppressed after the induction of inflammation through exercise. These results demonstrate that hyaluronic acid supplementation does act as an anti-inflammatory in skeletal muscle tissue, but does not have long-term suppressive effects when inflammation does occur.

**Abstract:**

Following strenuous exercise, skeletal muscle experiences an acute inflammatory state that initiates the repair process. Systemic hyaluronic acid (HA) is injected to horses routinely as a joint anti-inflammatory. To gain insight into the effects of HA on skeletal muscle, adult Thoroughbred geldings (n = 6) were injected with a commercial HA product weekly for 3 weeks prior to performing a submaximal exercise test. Gluteal muscle (GM) biopsies were obtained before and 1 h after exercise for gene expression analysis and HA localization. The results from RNA sequencing demonstrate differences in gene expression between non-injected controls (CON; n = 6) and HA horses. Prior to exercise, HA horses contained fewer (*p* < 0.05) transcripts associated with leukocyte activity and cytokine production than CON. The performance of exercise resulted in the upregulation (*p* < 0.05) of several cytokine genes and their signaling intermediates, indicating that HA does not suppress the normal inflammatory response to exercise. The transcript abundance for marker genes of neutrophils (*NCF2*) and macrophages (*CD163*) was greater (*p* < 0.05) post-exercise and was unaffected by HA injection. The anti-inflammatory effects of HA on muscle are indirect as no differences (*p* > 0.05) in the relative amount of the macromolecule was observed between the CON and HA fiber extracellular matrix (ECM). However, exercise tended (*p* = 0.10) to cause an increase in ECM size suggestive of muscle damage and remodeling. The finding was supported by the increased (*p* < 0.05) expression of *CTGF*, *TGFβ_1_*, *MMP9*, *TIMP4* and *Col4A1*. Collectively, the results validate HA as an anti-inflammatory aid that does not disrupt the normal post-exercise muscle repair process.

## 1. Introduction

Hyaluronic acid (HA), alternatively referred to as hyaluronan or hyaluronate, is a non-sulfated glycosaminoglycan (GAG) that is found in the extracellular matrix (ECM) of most body tissues. The GAG is marketed as an injectable drug for degenerative joint diseases where it acts to improve synovial fluid viscosity and lubrication properties in horses, people and dogs [1]. The injection of HA at pharmacological doses can alleviate pain and act as an anti-inflammatory [2]. The underlying mechanism of action of the GAG may include the suppression of the Toll-like receptor–nuclear factor kappa B (TLR-NFkB) signaling axis in macrophages and neutrophils, thus preventing inflammatory cytokine production [3,4,5]. Recent discoveries with nanoparticles embedded with HA and biomaterials constructed from HA polymers offer promise as soft-tissue repair aids that may translate to chemotherapeutic approaches for cancer cell targeting [6]. Xenografts of bovine bone devoid of donor cells and growth factors demonstrate improved host osteoblast viability and migration, as well as augmented angiogenic properties, following surface coating of the engraftment material with HA [7,8]. The ability to capture the anti-inflammatory properties of HA while supporting wound and damage repair remains challenging.

The production of hyaluronate in skeletal muscle is developmentally regulated through the temporal and spatial expression of the hyaluronate synthetase (HAS) 1-3 enzymes [9]. The peak expression of HAS2 appears during fetal myogenesis in the mouse, where it facilitates myoblast differentiation and myofiber formation. In addition to serving as a positive effector of fusion in the embryo, HA guides myoblast migration along the ECM surface through its receptor, RHAMM [10]. The concept of directed migration through HA can be extended to satellite cells, the postnatal skeletal muscle stem cells responsible for muscle repair, regeneration and fiber hypertrophy [11]. Following synergist ablation and compensatory hypertrophy in muscle, macrophages express abundant amounts of HAS2, leading to HA synthesis and its insertion into the surrounding basal lamina [12]. The local deposition of the GAG by macrophages may serve to direct the muscle stem cell to points of focal damage along the fiber while preserving their stem-like properties [13]. The interplay among immune cells, satellite cells and fibroblasts maintains the muscle fiber niche and supports homeostatic control of the tissue. 

Human and equine skeletal muscle experiences damage following strenuous exercise, as indicated by increased creatine kinase, myoglobin and lactate dehydrogenase within an hour of completion of the activity [14,15,16]. Damage resolution begins with establishment of an inflammatory environment within the muscle. Neutrophils within the muscle secrete TNFα and IL1β that establish the inflammatory condition and promote the recruitment of additional systemic neutrophils and macrophages (M1) that remove the necrotic tissue [17]. The M1 macrophage also secretes TNFα, IL6 and IL1β, which further maintains the local inflammatory milieu and facilitates muscle repair [18,19]. The numbers of phagocytic M1 cells decline precipitously with a coincident increase in the numbers of anti-inflammatory polarized M2 macrophages within the first 2 days [20]. The phenotypic switch of the macrophages is mediated by IL10, which also promotes myoblast differentiation and fiber formation [21]. The disruption of either phase of the acute inflammatory response is detrimental to muscle damage repair and recovery. The inhibition of leukocyte invasion, suppression of TNFα activity or perturbation of macrophage polarization prolongs repair [22,23,24,25]. Thus, post-exercise recovery aids that modify the inflammatory response may delay muscle repair.

The systemic delivery of HA prior to competitions is gaining popularity in the lay equestrian community, possibly due to its nociceptive properties [26]. Because of its noted anti-inflammatory properties, the hypothesis that HA injection would suppress inflammatory gene expression in skeletal muscle and impede the immediate post-exercise recovery period was tested in adult horses.

## 2. Materials and Methods

All animal protocols and procedures were reviewed and approved by the Virginia Tech Institutional Animal Care and Use Committee (22-170).

### 2.1. Animal Care and Groupings

Twelve mature unfit Thoroughbred geldings with a mean age of 6.83 ± 2.04 years and body weight (BW) of 547 ± 40 kg were housed in groups of three in dry lots of equal size (0.2 ha) with ad libitum access to water and minerals. All horses were clinically normal, sound and had no history of joint disease or trauma. The horses received mixed-grass hay at 1.8% of BW and were fed a commercial feed (2.2 kg, Ultium Competition, Land O’Lakes, Arden Hills, MO, USA) split evenly into two meals daily. The daily intake was sufficient to meet the nutritional needs of adult sedentary horses [27].

### 2.2. Treatment and Exercise 

The horses (n = 6) were injected with 40 mg of sodium hyaluronate purified from the capsule of Streptococcus spp. (HA; Legend, Boehringer Ingelheim, Duluth, GA, USA) into the jugular vein each week for 3 weeks, according to the manufacturer’s recommendations. Control horses (CON, n = 6) received no intravenous injection. Two weeks after the final injection (d 35 of the experiment) and 2 h post-prandial, all horses were fitted with a heart rate monitor (Polar Equine H10, Kempele, Finland) and performed a submaximal exercise test on a free run exerciser (EquiGym LLC, Paris, Kentucky). The exercise test parameters consisted of four repetitions of 6 min of walk (2 m/s), 14 min of trot (4 m/s), and 10 min of canter (8 m/s) with a change in direction between reps [28,29]. 

### 2.3. Muscle Biopsies and Tissue Preparation 

Skeletal muscle biopsies were taken from the gluteus medius (GM) of all horses from alternating limbs on d 0 prior to the first Legend injection (left GM), d 34 (left GM), and d35 (right GM) one hour after completion of the submaximal exercise test. The horses were sedated with xylazine (Bimeda-MTC Animal Health, Cambridge, ON, Canada) and the hair above the center of the GM was shaved and cleaned with chlorohexidine (Nolvasan scrub, Zoetis, Kalamazoo, MI, USA) and 70% ethanol (*v*/*v*) in water. The biopsy site was anesthetized with a subcutaneous injection of 2% lidocaine (VetOne, Biose, ID, USA) and a surgical scalpel blade was used to pierce the skin. A 10-gauge vacuum-assisted biopsy needle (EleVation, BD Biosciences, Franklin Lakes, NJ, USA) was inserted into the initial incision perpendicular to the muscle and 5 cm deep. The tissue samples were washed with phosphate-buffered saline (PBS) prior to embedding in optimal cutting temperature media (OCT; Thermofisher, Waltham, MA, USA) and freezing or flash freezing in liquid nitrogen. All samples were stored at −80 °C until needed. 

### 2.4. Muscle Cryosectioning and Staining

Cryosections (15 µm) affixed to glass slides were washed three times with PBS and blocked with 1% bovine serum albumin (BSA) in PBS for 30 min at room temperature. Following a brief rinse with PBS, the tissue sections were incubated at room temperature with a biotinylated recombinant hyaluronan-binding protein (250 µg/mL; AMS.HKD-BC41; AMSBIO, Cambridge, MA, USA) reconstituted in sterile water and diluted (10 µg/mL) in PBS for 1 h. The sections were washed and stained with Streptavidin Alexa Fluor 568 conjugate (2 µg/mL), Wheat Germ Agglutinin Oregon Green™ 488 conjugate (2.5 µg/mL, Thermofisher) and Hoechst 33342 (1 µg/mL, Thermofisher) in PBS for 1 h at room temperature. The slides were mounted with 10% glycerol and stored at 4 °C until imaging. 

### 2.5. Epifluorescent Imaging

Representative microscope images (n = 12) of stained skeletal muscle cryosections (n = 2 per horse) were captured at 100- or 200-fold magnification using an epifluorescent microscope (ECHO Revolve, San Diego, CA, USA). Digital images were annotated using the manufacturer-supplied software. The total fluorescence was measured by taking the average fluorescence intensity and multiplying it by the area being measured. The ratio of HABP-AlexaFluor594:WGA-AlexaFluor488 was calculated as the relative amount of HA within the basal lamina (BL). Images at 100-fold magnification were used to measure the average cross-sectional area of muscle fibers and the size of the BL. The cross-sectional area was measured by outlining the fibers (n = 30 per horse). The width of the BL was measured by measuring the distance of the green fluorescent band between two fibers (n = 48 measurements per horse). 

### 2.6. Total RNA Isolation

Approximately 500 mg of frozen skeletal muscle tissue was homogenized in TRIzol (Invitrogen, Carlsbad, CA, USA) using a handheld immersion disperser (Kinematic Polytron, Thomas Scientific, Swedesboro, NJ, USA). The homogenized tissue was incubated at room temperature for 20 min prior to the addition of chloroform (20% final volume). The aqueous layer was collected via centrifugation and the total RNA precipitated by the addition of isopropanol. Following centrifugation, the RNA pellet was resuspended in sterile water and further purified using spin columns (PureLink RNA kit, Thermofisher). The total RNA was eluted into sterile water and quantified by spectrophotometry (NanoDrop, Thermofisher). RNA integrity was measured by capillary electrophoresis and all RIN values were greater than 8.0. The total RNA was stored at −80 °C until use.

### 2.7. RNA Sequencing and Bioinformatics

All RNA sequencing was performed by the Novogene Corporation (Durham, NC, USA). In brief, poly A mRNA was isolated and used for the construction of cDNA libraries. The quantified libraries were sequenced on an Illumina platform and pair-end reads generated. Raw reads were processed through fastp to remove adapters and low-quality reads. Cleaned reads were aligned to the *Equus caballus* genome with Hisat2. FeatureCounts v1.5.0-p3 was used to count the read numbers mapped to each gene. The fragments per kilobase of transcript sequence per millions (FPKM) of the base pairs sequenced was calculated based on the length of the gene and the read counts mapped. Differential expression analysis was performed using the DESeq2 R package (1.20.0) and *p*-values were adjusted using the Benjamini–Hochberg method. Gene Ontology (GO) enrichment analysis of differentially expressed genes was performed with clusterProfiler R with corrected gene length bias. GO terms with corrected *p* < 0.05 were considered significantly enriched. A similar approach was used for KEGG enrichment analysis. Principle component analysis was performed using the pre- and post-exercise gene expression values. The results demonstrated that two libraries failed to segregate within the respective exercise groups and were removed from further analyses.

### 2.8. Statistical Analysis

The experiment was designed as a 2 × 2 factorial with HA injection and exercise as the primary variables. The analysis was performed as a two-way ANOVA with repeated measures for the effects of treatment, exercise, and their interaction. Sidak’s multiple comparison mean separation was performed where appropriate using GraphPad Prism (v.10; Boston, MA, USA). For selected exercise genes, the data were analyzed by paired *t*-test. Significance was established as *p* ≤ 0.05 and a tendency as *p* ≥ 0.06 ≤ 0.10). 

## 3. Results

Libraries were constructed from the total RNA isolated before and after exercise from both the CON and HA-treated horses. The average total reads for the samples was approximately 49 million and ranged from 40,936,546 to 77,200,234 bp. More than 85% of the sequencing reads mapped to the *Equus caballus* genome, with 75.2 ± 0.28% mapping to exons and 8.5 ± 0.20% mapping to introns. The remaining 16.3 ± 0.19% of the sequencing reads represent intergenic regions. 

**Figure 1:**
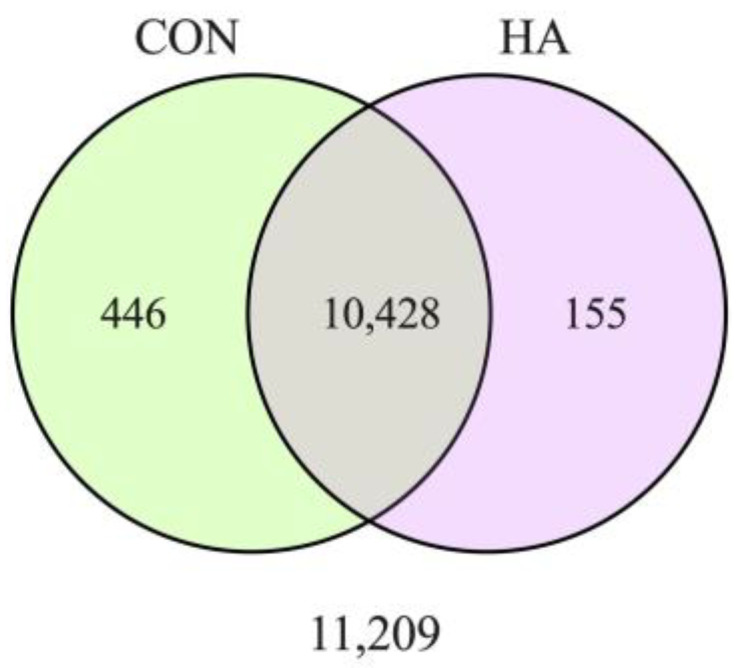
DEGs were identified between CON and HA treatment groups. The majority of genes were common between groups. Here, 155 genes were unique to HA and 446 genes were unique to CON.

The transcript abundance between CON and HA was compared to evaluate the differential gene expression (DEG). Genes with fewer than one count per million reads in three or more of the samples were removed from the dataset. A total of 11,029 genes were quantified and used to construct a Venn diagram that demonstrates that 10,428 of the genes are common between the sample groups, with 155 genes being unique to HA and 446 genes being unique to CON (Figure 1). 

**Figure 2:**
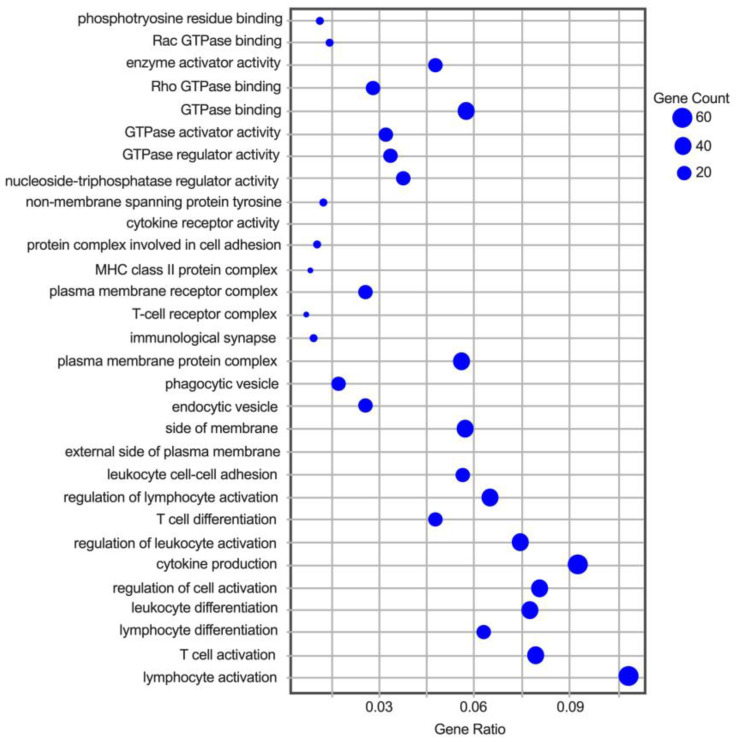
Gene ontology (GO) terms associated with inflammatory DEGs affected by HA treatment. Enrichment analysis was performed with clusterProfiler and the GO terms with the greatest numbers of DEGs are shown. The gene count histogram is scaled to size, with the largest blue dots containing at least 60 DEGs and the smallest fewer than 20 DEGs per GO term.

**Figure 3:**
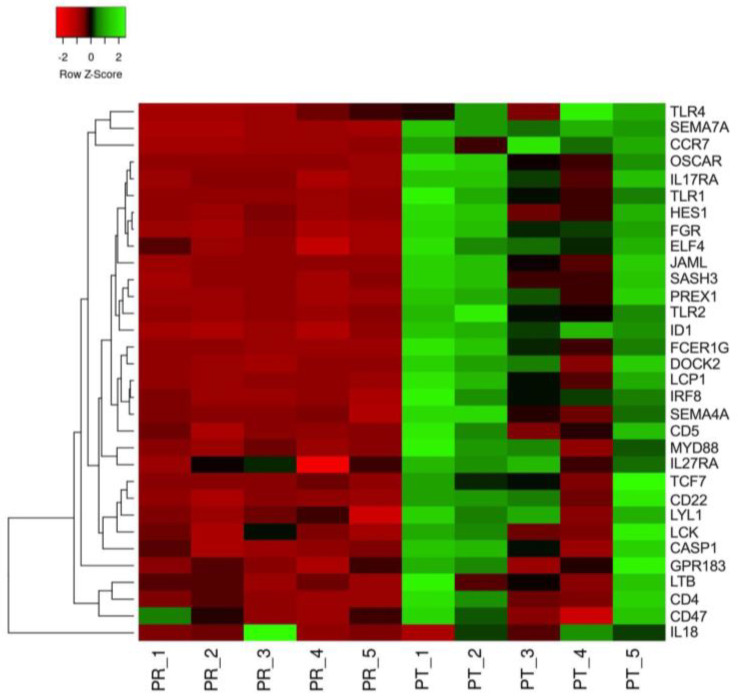
Inflammatory DEGs downregulated by HA are not refractile to exercise-induced stress. A heat map was constructed comparing pre- (PR_1-5) and post-exercise (PT_1-5) reads per kilobase million for selected DEGs (right column) in HA-treated horses. Row Z-scores range from −2-fold lower (red) to +2-fold greater (green). Dendrogram denotes average linkage between genes with distance measured by Spearman rank correlation.

Gene ontology (GO) terms were assigned to the DEGs and analyzed for clusters of genes segregated by known functions. The greatest number of DEGs was associated with T-cells and the inflammatory response (Figure 2). The specific genes within the lymphocyte activation and cytokine production GO families that were downregulated by HA treatment included *IL17RA*, *OSCAR*, *LYL1* and *TLR1*, *2*, and *4*. Because the initial post-exercise response requires acute inflammation, a heat map comparing pre- and post-exercise reads per kilobase million (RPKM) for selected DEGs within the GO cluster was constructed to visualize the response to exercise (Figure 3). The map demonstrates a global increase in expression for the majority of the genes associated with inflammation, with different magnitudes of responses being apparent amongst the horses following the stressor test. These results indicate that the majority of the inflammatory genes are not irreversibly downregulated. 

**Figure 4:**
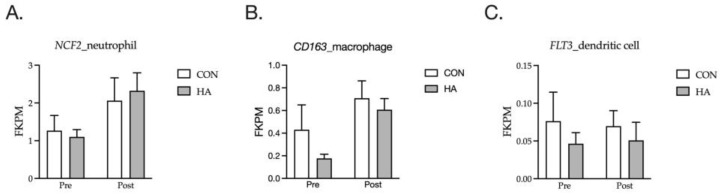
Neutrophil and macrophage invasion was apparent in the muscle tissue at 1 h post-exercise. An increased expression of cell markers *NCF2* ((**A**); *p* = 0.05) and *CD163* ((**B**); *p* < 0.01) was observed. No change in dendritic cell marker *FLT3* (*p* > 0.05) was observed (**C**).

Marker genes for neutrophils, macrophages and dendritic cells were examined for their relative expression during the acute inflammatory period post-exercise (Figure 4). As previously described, neutrophil invasion occurred in the post-exercise period at 1 h, as indicated by the increased expression of *NCF2* and *ELANE* (*p* = 0.05 and *p* = 0.07, respectively). The macrophage expressed gene, *CD168*, increased (*p* < 0.01) with exercise, but no change was observed (*p* > 0.05) in the dendritic cell marker *Flt3*.

**Figure 5:**
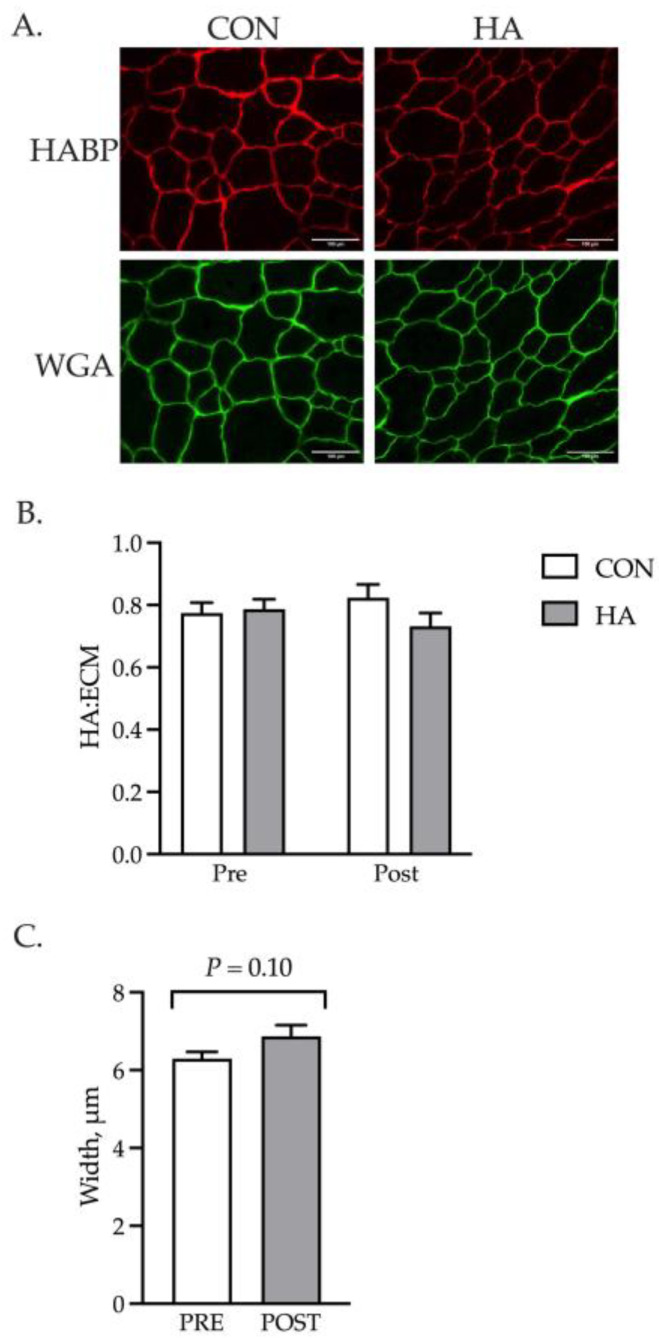
Exercise affects the structure of the basal lamina. HA was localized to the basal lamina (WGA) of muscle fibers with HABP (**A**). The ratio of HABP:WGA was calculated and used as an estimate of basal lamina composition (HA:ECM) (**B**). The width of the basal lamina was measured throughout the GM samples of HA and CON horses before and after exercise (**C**). HA injection did not alter the relative quantity of HA within the ECM of muscle fibers before or after exercise. Exercise tended to increase (*p* = 0.10) the size of the ECM.

**Figure 6:**
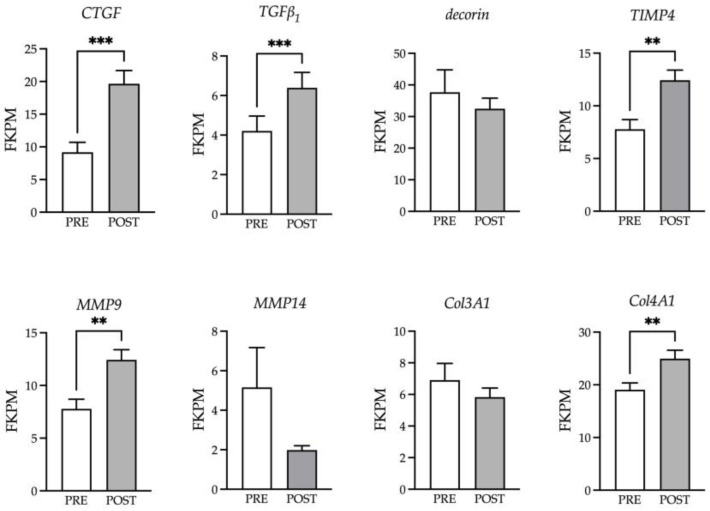
Genes associated with ECM remodeling are upregulated by exercise. Select genes for growth factors (*CTGF*, *TGFβ_1_*, *decorin*), ECM proteases (*MMP9*, *MMP14*, *TIMP4*) and collagens (*Col3A1*, *Col4A1*) involved in remodeling were examined before (PRE) and 1 h after (POST) exercise. ** denotes significance at *p* < 0.01; *** denotes significance at *p* < 0.001.

The localization of HA to the basal lamina of the muscle fiber was examined with HABP using cryosections obtained before and after exercise (Figure 5A). The ratio of HA to ECM was calculated for the CON and HA samples as a proxy for relative abundance. No differences were observed for the amount of HA between the groups (Figure 5B). While no increased HA deposition was noted, exercise tended to increase (*p* = 0.10) the width of the ECM (Figure 5C). Coincident with the larger size, a greater abundance of genes involved with matrix remodeling was detected and included *CTGF*, *TGF-β1*, *MMP9* and *Col4A1* (Figure 6). 

## 4. Discussion

Hyaluronic acid (HA) is a naturally occurring glycosaminoglycan (GAG) that, when supplemented into the body, has anti-inflammatory effects and leads to improved lameness scores in horses [30]. Following intra-articular injection in people, HA acts as an anti-inflammatory in synovial fluid by reducing IL-6, IL-1B and TNFα production through CD44-receptor-mediated signals [3,31]. A recent study in mice suggested that HA serves a similar role in skeletal muscle by protecting the tissue-resident muscle stem cells from the cytokine barrage that occurs in response to damage [32]. The results from the studies herein support an anti-inflammatory role for the GAG in muscle by reducing the expression of numerous genes associated with T-lymphocyte recruitment, activation and function. The diminished expression of these genes in the sedentary state may offer advantages for the global health of the horse. Horses experiencing obesity and metabolic syndrome, both inflammatory diseases, may benefit from systemic HA treatment through its ability to reduce cytokine levels and inflammatory signaling through the Toll-like receptor (TLR) system [33,34]. Isolation and transcriptome analysis of adipose deposits from horses receiving HA would provide insight into the role of the GAG as a master regulator of tissue inflammation.

Endogenous HA localized within the ECM originates from the coupling of monomers of UDP-glucuronic acid and UDP-N-acetylglucosamine by HA synthases (HAS) [35]. The elongation of the extracellular linear polysaccharide chains leads to diverse sizes of HA ranging from 10^5^ to 10^7^ daltons. The size of the molecule influences its bioactivities with lower Mr forms associated with the inflammatory state and larger isoforms regarded as anti-inflammatory [36]. Although the average size of the HA molecule supplied by Legend is unknown, it is likely similar to other common HA drugs used in horses that range in size from 2.1 MDa (Hyvisc) to 3.5 MDa (Hylartin-V) [37]. Our results demonstrate that the ectopic delivery of these large biomolecules does not result in their insertion into the basal lamina of the muscle fiber. This is not surprising, as others report the rapid clearance of HA from the blood following injection of a similar dose into adult horses and mice with no accumulation in the organs or joints [38,39]. The indirect actions of the GAG may be mediated, in part, through the inhibition of the innate immune response within the tissue. Human and mouse myoblast and myotube cultures express TLR1-7, but not TLR8 [40,41]. The treatment of muscle cultures in vitro with LPS or palmitate results in the upregulation of *IL6* through signals mediated by TLR2 and TLR4, respectively [41,42,43]. *Toll-like receptors 1*, *2* and *4* were lower in the pre-exercise gluteal muscle from HA-treated horses in comparison to their post-exercise counterpart. The diminished expression of *TLR1* and *2* in the muscle fiber leading to the suppression of intracellular signals that stimulate an inflammatory response is an attractive mechanism of action for HA. 

Skeletal muscle damage repair and regeneration requires an initial inflammatory state that facilitates neutrophil and macrophage invasion for the targeted removal of damage and necrotic material [17]. The depletion of the invading macrophage population results in smaller muscle fibers and increased muscle adiposity following a myonecrotic insult, demonstrating the importance of the macrophage to the initial inflammatory response [44,45]. The polarization of the M1 macrophage to the M2 state denotes the transition from proinflammation to that of an anti-inflammatory condition that supports the fusion of satellite cells to the adjacent damaged fiber [46]. Because exercise causes microdamage to the fiber that requires repair, it is critical that the normal progression of inflammation through resolution occurs. The suppression of muscle inflammatory gene expression by HA was not irreversible. The heat map of the selected genes associated with leukocyte activation revealed a relative two-fold increase in expression following the exercise stressor. Marker genes for neutrophils and macrophages were upregulated within the first hour post-exercise, consistent with a normal reparative process. Although the complete timeline of inflammation resolution and muscle repair was not measured, no differences in CD168 expression between the CON and HA-treated horses post-exercise indicates that similar numbers of macrophages are present and available for the polarization and subsequent support of satellite cell fusion and fiber repair. However, caution is warranted as no direct measure of immune cell infiltration was measured. Equally important is the variable response of individual horses amongst the inflammatory genes. Although a power analysis revealed that the original study contained sufficient numbers of horses to observe statistically significant differences in exercise target genes, the large variation in the inflammatory gene expression patterns suggests that future efforts should use more subjects. 

Damage to skeletal muscle tissue causes an upregulation of genes associated with myofiber ECM remodeling [47]. Within 30 min of an acute bout of exercise, connective tissue growth factor (CTGF) expression is increased in skeletal muscle and remains elevated through the initial inflammatory period [48,49]. A similar expression pattern is reported for *TGF-β_1_* and is independent of exercise type (concentric, eccentric, isometric) [49]. These members of the TGF-β superfamily drive the transcription of several collagens and proteoglycans that maintain the integrity of the fiber basal lamina and allow for adaptative responses to exercise training [50]. The modest increase in ECM width is unlikely to be biologically relevant, but does supply additional information that the basal lamina of the fiber is disrupted and part of the immediate early repair process within the muscle. A linear pathway from *CTGF* and *TGF-β_1_* to the increased expression of *TIMP4*, *MMP9* and *Col4a1* that is active within the first hour may be important to modify the elasticity of the matrix and facilitate the movement of macrophages and neutrophils within the tissue. Crosstalk between macrophages, fibroblasts and satellite cells is crucial to effective ECM remodeling. The secretion of MMP14 from macrophages underlies the hypertrophic response of muscle fibers following synergist ablation [51]. The metalloproteinase, ADAMTS1, released from macrophages following muscle damage increases the number of mitotically active muscle stem cells [52]. Macrophage secretion of TGF-β1 prevents follistatin release from niche-localized fibroblasts, thus providing a suitable environment for muscle fiber regeneration and fiber growth [53]. Our results suggest that similar intercellular communication channels exist in equine muscle, leading to ECM perturbations. However, the interconnections between the cells and their transcriptomes require further evaluation to fully understand the complexity of the early repair response in equine muscle. 

## 5. Conclusions

Sodium hyaluronate acts as an anti-inflammatory in equine skeletal muscle through the suppression of basal inflammatory gene expression. Genes involved in leukocyte activation and cytokine production are not irreversibly downregulated and exhibit increased expression following an exercise stressor. Expression analysis pre- and post-exercise reveals a complex cellular network replete with monocytes, macrophages, fibroblasts and myogenic precursors. The interactions between these cells following exercise and during metabolic insults and disease require further elucidation. 

## Data Availability

The data presented in this study are available as BioProject PRJNA1007689 through the National Center for Biotechnology Information.
